# The Interplay Between Plasma Hormonal Concentrations, Physical Fitness, Workload and Mood State Changes to Periods of Congested Match Play in Professional Soccer Players

**DOI:** 10.3389/fphys.2020.00835

**Published:** 2020-07-21

**Authors:** Karim Saidi, Abderraouf Ben Abderrahman, Daniel Boullosa, Grégory Dupont, Anthony C. Hackney, Benoit Bideau, Thomas Pavillon, Urs Granacher, Hassane Zouhal

**Affiliations:** ^1^Movement, Sport, Health and Sciences Laboratory (M2S), University of Rennes 2, Rennes, France; ^2^Higher Institute of Sport and Physical Education of Ksar Said, University of Manouba, Tunis, Tunisia; ^3^Faculty of Health Sciences, University of Brasilia, Brasília, Brazil; ^4^Real Madrid Football Club, Madrid, Spain; ^5^Department of Exercise and Sport Science, The University of North Carolina at Chapel Hill, Chapel Hill, NC, United States; ^6^French Football Federation (FFF), Paris, France; ^7^Division of Training and Movement Sciences, University of Potsdam, Potsdam, Germany

**Keywords:** training, hormones, overtraining, overreaching, recovery

## Abstract

**Background:**

The regular assessment of hormonal and mood state parameters in professional soccer are proposed as good indicators during periods of intense training and/or competition to avoid overtraining.

**Objective:**

The aim of this study was to analyze hormonal, psychological, workload and physical fitness parameters in elite soccer players in relation to changes in training and match exposure during a congested period of match play.

**Methods:**

Sixteen elite soccer players from a team playing in the first Tunisian soccer league were evaluated three times (T1, T2, and T3) over 12 weeks. The non-congested period of match play was from T1 to T2, when the players played 6 games over 6 weeks. The congested period was from T2 to T3, when the players played 10 games over 6 weeks. From T1 to T3, players performed the Yo-Yo intermittent recovery test level 1 (YYIR1), the repeated shuttle sprint ability test (RSSA), the countermovement jump test (CMJ), and the squat jump test (SJ). Plasma Cortisol (C), Testosterone (T), and the T/C ratio were analyzed at T1, T2, and T3. Players had their mood dimensions (tension, depression, anger, vigor, fatigue, confusion, and a Total Mood Disturbance) assessed through the Profile of Mood State questionnaire (POMS). Training session rating of perceived exertion (sRPE) was also recorded on a daily basis in order to quantify internal training load and elements of monotony and strain.

**Results:**

Significant performance declines (T1 < T2 < T3) were found for SJ performance (*p* = 0.04, effect size [ES] ES_1__–__2_ = 0.15−0.06, ES_2__–__3_ = 0.24) from T1 to T3. YYIR1 performance improved significantly from T1 to T2 and declined significantly from T2 to T3 (*p* = 0.001, ES_1__–__2_ = 0.24, ES_2__–__3_ = −2.54). Mean RSSA performance was significantly higher (*p* = 0.019, ES_1__–__2_ = −0.47, ES_2__–__3_ = 1.15) in T3 compared with T2 and T1. Best RSSA performance was significantly higher in T3 when compared with T2 and T1 (*p* = 0.006, ES_2__–__3_ = 0.47, ES_1__–__2_ = −0.56), but significantly lower in T2 when compared with to T1. T and T/C were significantly lower in T3 when compared with T2 and T1 (T: *p* = 0.03, ES_3__–__2_ = −0.51, ES_3__–__1_ = −0.51, T/C: *p* = 0.017, ES_3__–__2_ = −1.1, ES_3__–__1_ = −1.07). Significant decreases were found for the vigor scores in T3 when compared to T2 and T1 (*p* = 0.002, ES_1__–__2_ = 0.31, ES_3__–__2_ = −1.25). A significant increase was found in fatigue scores in T3 as compared to T1 and T2 (*p* = 0.002, ES_1__–__2_ = 0.43, ES_2__–__3_ = 0.81). A significant increase was found from T1 < T2 < T3 intension score (*p* = 0.002, ES_1__–__2_ = 1.1, ES_2__–__3_ = 0.2) and anger score (*p* = 0.03, ES_1__–__2_ = 0.47, ES_2__–__3_ = 0.33) over the study period. Total mood disturbance increased significantly (*p* = 0.02, ES_1__–__2_ = 0.91, ES_2__–__3_ = 1.1) from T1 to T3. Between T1-T2, significant relationships were observed between workload and changes in T (*r* = 0.66, *p* = 0.003), and T/C ratio (*r* = 0.62, *p* = 0.01). There were significant relationships between performance in RSSA_best_ and training load parameters (workload: *r* = 0.52, *p* = 0.03; monotony: *r* = 0.62, *p* = 0.01; strain: *r* = 0.62, *p* = 0.009). Between T2-T3, there was a significant relationship between Δ% of total mood disturbance and Δ% of YYIR1 (*r* = −0.54; *p* = 0.04), RSSA_best_ (*r* = 0.58, *p* = 0.01), SJ (*r* = −0,55, *p* = 0.01), T (*r* = 0.53; *p* = 0.03), and T/C (*r* = 0.5; *p* = 0.04).

**Conclusion:**

An intensive period of congested match play significantly compromised elite soccer players’ physical and mental fitness. These changes were related to psychological but not hormonal parameters; even though significant alterations were detected for selected measures. Mood monitoring could be a simple and useful tool to determine the degree of preparedness for match play during a congested period in professional soccer.

## Introduction

Contemporary elite soccer players are exposed to a high number of matches and confronted with a large number of fixtures throughout the season, including league cup and other competitions. With fixture, we refer to a schedule of matches that a football club must play over a season (Carling et al., 2012; Nédélec et al., 2012). Moreover, elite soccer players often play competitive matches with only 2–3 days of recovery in between (Ekstrand et al., 2004; Krustrup et al., 2011; Rowsell et al., 2011; Arruda et al., 2015). This means that soccer players are continuously subjected to a variety of physical and psychological stressors from both, training and competition (Ekblom, 1986). As a consequence of the soccer-specific physiological demands, training programs should be specifically tailored and address physical qualities such as aerobic capacity, strength, power, and change-of-direction speed. Consequently, the goal of training is to provide adequate stimuli to induce soccer-specific adaptations that will improve physical performance over time. Maintaining or improving performance is not only determined by training, but also the capacity of bodily systems (e.g., neuromuscular system, endocrine system) to recover and regenerate after a period of intense training or after psychological stress caused by high number of competitions during a congested period. During congested periods, the dynamic homeostatic balance between anabolic and catabolic processes in the muscle can ultimately influence players’ physical fitness level (Moreira et al., 2016). The effects of soccer training on hematological variations (Saidi et al., 2019), immunological markers (Rebelo et al., 1998) muscle strength (Silva et al., 2011), physical performance (Dupont et al., 2010; Dellal et al., 2015), hormonal responses to single game (Thorpe and Sunderland, 2012), and long-term training have previously been reported. However, only a few studies exist that systematically tracked hormonal responses during different periods of competition over the course of a season (Silva et al., 2013). Indeed, biological and quantitative evaluations are now a common practice used for the evaluation of physiological responses during competition and training in many sports. A wide variety of biochemical, hematological, and physiological markers have been used for long-term monitoring of athletes (Heisterberg et al., 2013; Silva et al., 2013). In particular, cortisol (C) and testosterone (T) have been identified as reliable markers of training stress and could be considered two important hormones in the biochemical assessment of athletes (Adlercreutz et al., 1986; Hackney, 2020). In addition, the T/C ratio has additionally been used to evaluate soccer players’ anabolic/catabolic status (Urhausen et al., 1995). A decline in this ratio has previously been associated with the overtraining syndrome (Viru and Viru, 2001). Reflective of this study, Freitas et al. (2014) provided evidence that intensive periods of training may lead to an increase in salivary C concentrations in elite young soccer players. According to da Silva et al. (2011), 12-weeks of training with professional soccer players resulted in increased serum concentration of C with concomitant decreases in T and T/C ratio. Thus, hormonal parameters are sensitive to both, training periods that differ in terms of volume and intensity (da Silva et al., 2011), and frequency of matches during the competitive period (Silva et al., 2013). While the use of biochemical markers for long-term monitoring of exercise stress is widespread, the relationship between hormonal concentrations and physical performance standards during different periods of competitive season remains unclear. If the physical demands of training and competition are too great, one might assume that catabolic activities will predominate. If, however, the body is able to successfully cope with the demands, the anabolic metabolism can help maintain or improve the performance during different periods of the competitive season (Fry et al., 1993). However, many factors influence this delicate hormonal balance, not only training workloads, training schedules, competition factors but also psychological stress. The interplay of these factors can influence the overall physiological state and readiness of the player. Thus, Edwards et al. (2015) have shown that players with a positive mood state activated the parasympathetic nervous system and consequently cortisol secretion decreased. In contrast, negative mood states can activate the sympathetic nervous system, higher C secretion can take place. In this regard, physiological influences of stress and mood state may intervene and have rebound effects on physical fitness (Chennaoui et al., 2016). This emphasizes the necessity to assess the interplay of hormonal, psychological, and physical fitness in elite soccer players during different competitive periods of a soccer season.

Thus, in light of the above, the aim of this study was two-fold; (1) to examine elite soccer players’ fluctuations in hormonal, psychological, and physical fitness parameters during the competitive period of the season, including a period of congested match play; and, (2) to examine the inter-relationships between all of these parameters. It was hypothesized that hormonal and psychological parameters may be altered after the congested periods of competition, which, in turn, could cause negative changes in players’ physical fitness which would be evident through the associations between selected measures (Faude et al., 2011; Silva et al., 2013; Moreira et al., 2016).

## Materials and Methods

### Participants

At the beginning of the study, 24 male professional soccer players aged 19 to 22 years from the same soccer team playing in the first Tunisian soccer division participated in this study (body height: 177.0 ± 4.0 cm; body mass: 72 ± 5.2 kg; BMI = 22.7 ± 1.1 kg/m^2^).

Over the course of the study, a few players got injured and could therefore not complete all training sessions. Athletes who attended less than 85% of the scheduled training sessions and matches were excluded from the study. In total, 16 players were eligible for inclusion in this study. The monitored players included three central backs, three fullbacks, six midfielders, and four strikers. All were engaged in systematic soccer training and in national competitions for at least 8 years. The enrolled players had the longest match exposure (i.e., players who were most often in the starting line-up). Each participant provided a written consent to participate after being fully informed of all experimental procedures. The study was carried out in accordance with the latest version of the Declaration of Helsinki and it was approved by the local Ethics Committee of the Scientific Council of the University of Rennes 2, France and the medical staff of the soccer club (Jeunesse sportive Kairouannaise, JSK).

### Procedures

**FIGURE 1:**
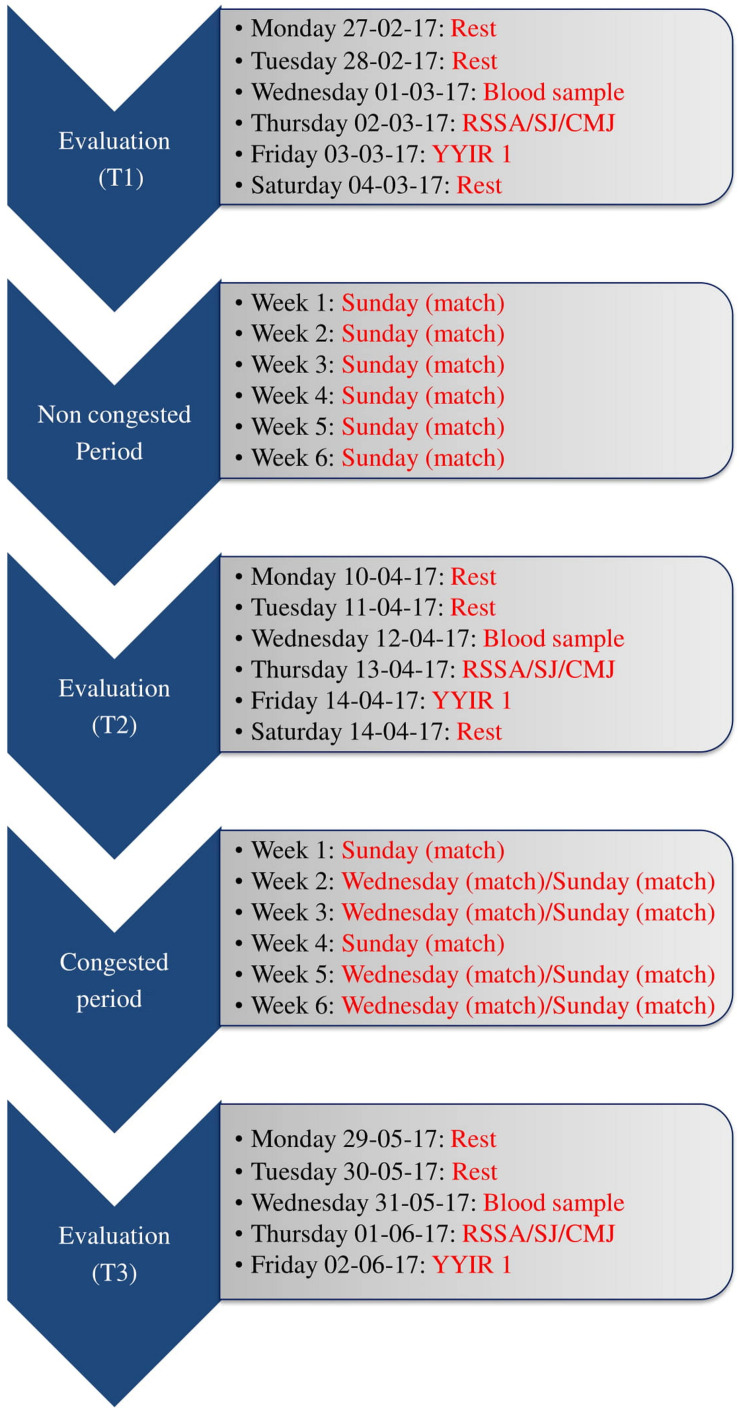
Experimental procedures. RSSA: Repeated Sprint Shuttle Ability. YYIR 1: Yo-Yo intermittent recovery test level 1. SJ: Squat Jump. CMJ: Countermovement Jump test.

Players’ evaluations started 6 weeks after the beginning of the competitive period following a winter break. During this period, players trained five times per week and played one match a week. All players were evaluated three times during the study; i.e., T1, T2, and T3. T1 was scheduled in the middle of the non-congested period (week 0; early March). T2 was at the end of the non-congested period (before the congested period; week 6; mid-April). T3 was completed after the congested period (week 12; late May until early June – after the season) (see Figure 1).

The testing of hormonal parameters and physical fitness were performed over 3 days during the T1, T2, and T3 test sessions. On the first day, blood samples were collected to determine resting hormonal parameters. On the second day, participants performed three physical fitness tests in the following order: squat jump (SJ), countermovement jump (CMJ) and the repeated shuttle sprint ability test (RSSA). Rest between tests amounted to 10 min and it was measured using a chronometer (lucky Stone professional digital stopwatch timer XL-0.13). Thus, players had sufficient time to recover from the tests.

On the third test day, participants performed the Yo-Yo intermittent recovery test level 1 (YYIR1). All physical fitness tests were performed in the afternoon, 3 h after taking a standard light meal. The applied physical fitness tests were familiar to all players because they are regularly conducted during the soccer season. In order to minimize diet-induced performance changes, players were asked to standardize and follow the same nutritional plan 24 h before each test session. Psychological parameters were also assessed three times during the study using the profile of mood state questionnaire (POMS) (McNair et al., 1971). The questionnaire was administered the day in which the blood samples were collected. Session ratings of perceived exertion (sRPE; Borg scale) were also recorded on a daily basis in order to quantify training impulse (TRIMP = sRPE × time in minutes), monotony, and strain (Foster, 1998).

### Training Program During the Non-congested and Congested Competitive Periods

Overall, 16 matches were played during the 12 weeks intervention period. Figure 1 summarizes the match distributions during the non-congested and congested periods of match play. Six games were played during the 6 weeks of the non- congested period, and 10 games were played during the 6 weeks of congested match play. Number and duration of training sessions over the course of the study were the same for all players and amounted to 56. The duration of each training session was 90 min for all players. Training sessions were performed during the afternoon (3:30 to 5:00 PM).

All training sessions were preceded by a 5–15 min standardized warm-up. The 6 weeks non-congested period contained three training sessions per week, which were conducted on Tuesday, Wednesday, Thursday, Friday, and Saturday. In addition, one match was played per week on Sundays. During weeks 7 and10 of the congested match play period, three training sessions were scheduled per week and performed on Tuesday, Wednesday, Thursday, Friday, and Saturday. During weeks 8-9-11-12, four training sessions were executed per week on Monday, Tuesday, Thursday, and Friday. In addition, two matches were played per week on Wednesdays and Sundays. A full recovery day (day off) was always provided on Saturday (see Table 1).

**TABLE 1:** Training programs during the non-congested and congested period of match play.

Day	Monday	Tuesday	Wednesday	Thursday	Friday	Saturday	Sunday
Weekly program during the non- congested period and during the period of congested match play (when playing one match per week). Week 1 to week 6, and weeks 7-10	Full recovery	Warm up, (15 min) Technical training (30 min) Low-to-moderate intensity aerobic exercise, (25 min) Small-sided games, (15 min)	Warm up, (15 min) Strength training, (25 min) Tactical training, (30 min) Reduced game, (15 min)	Warm up, (15 min) Tactical training, (35 min) Speed training over short distances, (20 min) Small-sided games, (15 min)	Warm up, (15 min) Technical training, (30 min) Speed training over long distances, (20 min) Soccer specific training, (25 min)	Warm up/technical training, (25 min) Speed training over short distances, (20 min) Soccer specific training, (30 min)	Match
Weekly program during the congested period of match play (when playing two matches per week). Weeks 8-9-11-12	Warm up, (15 min) Low-to-moderate intensity aerobic exercise, (30 min) Technical training, (35 min)		Match	Warm up, (15 min) Tactical training, (35 min) Speed training over short distances, (20 min) Small-sided games, (15 min)	Warm up, (15 min) Technical training, (30 min) Speed training over long distances, (20 min) Soccer specific training, (20 min)	Warm up/technical training, (25 min) Speed training over short distances, (20 min) Soccer specific training, (30 min)	Match

The weekly workload was evaluated from Monday to Sunday. The training program consisted of low-to-moderate intensity continuous running at 50–70% of the maximum heart rate (HRmax), high-intensity interval running at 90–100% of HRmax, specific soccer training drills according to the respective player’s position, speed training over 10-30-m (short) and 30-60-m (long) with and without a ball, tactical training and technical training (i.e., attack against defense in small fields), small sided games, and strength training.

The strength training program that was applied during the competitive period was different with regards to duration and intensity compared to that realized during the pre-season. In fact, the strength training sessions lasted 25 min per week and consisted of a circuit training designed primarily to improve muscle power. High velocity resistance exercises together with plyometrics were performed during circuit training.

Three substitute players had less game time than the rest of the cohort. Of note, training load of the substitution players was increased during the day after the match to reach a similar level compared with that of the players who participated in the whole match. Table 1 summarizes the training characteristics during the non-congested and congested periods of match play.

### Blood Sampling: Hormonal Assays

For each soccer player, blood samples were always taken at the same time of day (between 08:00- 10:00 AM) to determine hormonal status. A rest-recovery period of 12 h was scheduled the day before the samples were taken and participants followed an overnight fast. Blood glucose was analyzed with a chemistry analyzer (cobas c 111) to evaluate the nutritional status during the study. All evaluated players had blood glucose values ranging between 0.7 and 1.15 g/l which confirms the overnight fasting of soccer players. The test conditions were standardized. On the test day, blood samples (15 ml) were taken in a seated position via the median ante brachial vein into vacutainer tubes (Vacuette^®^, Greiner bioOne, France) without anti-coagulant and with EDTA (10 ml). The blood was immediately centrifuged at 3,000 rpm for 10 min to remove plasma. Plasma was stored at −80°C for further analyses. Plasma C and T were analyzed with VIDAS (ref.30418) commercial test kits (kit Vidas^®^, France) using the ELFA technique (enzyme linked fluorescent assay). All samples were taken and handled within a single laboratory.

### Physical Fitness Tests

#### Yo–Yo Intermittent Recovery Test Level1 (YYIRTL1)

The YYIRT1 was used to assess players’ ability to repeat high-intensity exercises (Bangsbo et al., 2008). For this purpose, cones were placed on 2 lines 20 m apart and 5 m behind the starting line. Players ran two 20 m shuttles following a beep signal, separated by a 10 s recovery period. The time between beeps decreased over the stages and players continued the test until they were unable to maintain the required pace for two successive beeps. Players were asked to place one foot either on or behind the 20-m line at the end of each shuttle. If a player did not reach the line in time, he was instructed to do so during the following run, in order to be able to continue the test. The final stage speed (km/h) that was achieved during the test and the total distance covered (m) were recorded for further analyses.

### Repeated Shuttle Sprint Test

Repeated Shuttle Sprint Ability (RSSA) was tested to assess players’ ability to cope with the specific sprinting demands of soccer. This test includes changes-of-direction tasks, which is a characteristic movement pattern in soccer. After a 15-min warm up, players completed 6 × 40 maximal sprints interspersed with 20 s of passive recovery. RSSA testing was conducted using an infrared photoelectric cell (Cell Kit Speed Brower, United States). The best sprint time was recorded and used for further analysis (RSSA_best_). In addition, the dependent variables included the mean time obtained over six sprints and the decrease in performance (The RSSA_decrement_ = ([RSA_mean_]/[RSA_best_] × 100) – 100)] (Impellizzeri et al., 2008). When in seated position, each player was pricked on the tip of the finger using a pricker. Adrop of blood was taken and applied to the strip (Lactate pro 2, H174B03L, Arkray factor, Inc., France). The portable lactate analyzer (Lactate Pro2, Matsport, France) determined the blood lactate level.

### Countermovement Jump (CMJ) and Squat Jump (SJ) Tests

The squat jump (SJ) started from a static semi-squatting position with knee angles at approximately ∼90°. After a short rest (2s) to avoid the storage of elastic energy, players performed the vertical jump. For the countermovement jump (CMJ), players started in a standing position and subsequently performed an explosive jump using a slow stretch-shortening cycle (SSC) with a ∼90° of knee flexion. Players were asked to jump as high as possible during both tests. Vertical jump height was evaluated using an optoelectric system (Opto-Jump Microgate – ITALY). Jump height was calculated according to the following equation: jump height = 1/8 × g × t^2^, where g is the acceleration due to gravity and t is the flight time (Prieske et al., 2013). All players performed three vertical jumps in the same order (i.e., SJ followed by CMJ), with hands akimbo and a 60s rest between each trial. The average of the best three trials was taken for further analysis (Claudino et al., 2017).

### Quantification of the Training Load

Total weekly training load was quantified on a daily basis using Rating of Perceived Exertion (RPE) assessment by means of an adapted Borg 0-10 scale (Foster, 1998). To ensure that the assessment of perceived effort reflected the entire session, data was collected approximately 15–20 min after each session. Training impulse (TRIMP) was calculated as sRPE × time (min) for each session. Monotony was calculated as the weekly average load divided by the standard deviation of the load. Strain was calculated as the training load multiplied by monotony (Foster et al., 2001). It has previously been suggested that these indices are negatively related with overreaching and/or overtraining (Foster et al., 2001).

### Psychological Tests

The Profile of Mood State questionnaire (POMS) was used to measure psychological adaptations to workloads during the experimental period. The POMS questionnaire provides measures of tension, depression, anger, vigor, fatigue, confusion, and a total mood disturbance. The total mood disturbance is obtained by adding all other measures and subtracting vigor (McNair et al., 1971). This questionnaire was used to monitor mood state and performance of athletes in a Team sports (Hoffman et al., 1999; Filaire et al., 2001).

### Statistical Analyses

Data were expressed as mean values and standard deviations (SD). All statistical tests were processed using SigmaStat 3.5 software (Systat, Inc., United States). Normal distribution of data was tested and confirmed using the Kolmogorov–Smirnov test. Paired *t*-tests for dependent samples were used to compare workload parameters, and Team performance between the congested and non- congested periods of match play. A one-way analysis of variance (ANOVA) for repeated measures was computed to determine differences between all parameters measured during the congested vs. non-congested period of match play. Relationships between parameters were assessed using Pearson’s product-moment correlation coefficient (*r*). The magnitude of correlation coefficients was considered as trivial (*r* < 0.1), small (0.1 < *r* < 0.3), moderate (0.3 < *r* < 0.5), large (0.5 < *r* < 0.7), very large (0.7 < *r* < 0.9), nearly perfect (0.9 < *r* < 1), and perfect (*r* = 1) (Hopkins and Burr, 2009). Statistical significance for all analyses was set at *p* < 0.05. The 95% confidence intervals (CI)and effects sizes (ES) were calculated to compare differences in mean values for all analyzed parameters during the 12 week-period of training (congested vs. non-congested period of match play). When calculating ES, pooled standard deviations (SD) were used since no control group was available (Cohen’s *d* = (M1-M2)/SD pooled). ES with values of 0.2, 0.5, and 0.8 were considered to represent small, medium and large differences, respectively (Cohen, 1988).

## Results

Over the 12-week experimental period, 16 players were able to complete the study requirements according to the previously described study design and methodology. The Mean ICC (Intraclass Correlation Coefficients) for the different parameters were presented in Table 2.

**TABLE 2:** Intraclass correlation coefficients.

Parameters	Mean Intraclass correlation coefficient (ICC)	95% confidence interval	
		Lower bound	Upper bound
Cortisol	0.74	0.40	0.90
Testosterone	0.83	0.61	0.93
T/C	0.82	0.59	0.93
YYIR1	0.48	0.51	0.78
RSSA_mean_,	0.61	0.15	0.84
RSSA_best_	0.69	0.34	0.89
RSSA_decrement_	0.34	–1.26	0.58
SJ	0.95	0.90	0.98
CMJ	0.98	0.77	0.95
TRIMPS	0.77	–1.20	0.93
Monotony	0.10	–0.20	0.08
Strain	0.004	–0.51	0.13
TMD	0.21	–0.54	0.67

*T/C: Testosterone/Cortisol ratio. YYIR1: Yo-Yo intermittent recovery test level 1. RSSA: Repeated Shuttle Sprint Ability. SJ: Squat Jump. CMJ: Countermovement Jump. TRIMP: Training Impulse. TMD: Total Mood Disturbance.*

### Training Workload

In Table 3, we presented data from workload analyses in periods of congested and non-congested match play. All training parameters were significantly different between comparisons.

**TABLE 3:** Training load parameters during the 12 weeks training period (congested vs. non-congested period of match play).

Training periods	From T1 to T2: Non-congested period	From T2 to T3: Congested period	*p*-Value	Effect size (ES)
TRIMP (A.U.)	8302.00 ± 1239.39	10092.96 ± 2362	<0.001*	0.50
Monotony (A.U.)	0.49 ± 0.07	1.87 ± 0.16	<0.001*	2.5
Strain (A.U.)	717.30 ± 174.60	10975.30 ± 2664.70	<0.001*	2.69

Data are presented as means ± SDs. *Significant difference between congested and non-congested period with *p* < 0.01. TRIMP: Training impulse, A.U: Arbitrary units.

### Physical Fitness Data and Team Performance

Performance test scores are illustrated in Table 4. The YYIR1, RSSA_mean_ and RSSA_best_, and SJ performance were significantly different in T3 after the congested period. In addition, the YYIR1 performance increased from T1 to T2 after the non-congested period. The RSSA_best_ were significantly lower in T2 as compared to T1. The CMJ, the RSSA_decrement_, and blood lactate after the RSSA test did not change significantly from T1 to T3.

**TABLE 4:** Changes in physical fitness.

Training periods	T1	T2	T3	*p*-Value	Effect size (ES) ES_1–2–_ES_2–3_
YYIR1 (m)	2536.2 ± 382.7	2635.5 ± 439.6	1650 ± 335.14	0.001*£	0.24_−2.54
RSSA_mean_ (s)	8.23 ± 0.33	8.07 ± 0.35	8.53 ± 0.46	0.019*	−0.47_1.15
RSSA_best_ (s)	7.70 ± 0.30	7.48 ± 0.48	7.77 ± 0.40	0.006*£	−0.56_0.47
RSSA_decrement_ (%)	7.01 ± 2.81	7.89 ± 4.71	6.05 ± 2.66	0.09	0.23_ 0.50
[Lac]_RSSA_ (mM/L)	18.03 ± 2.79	18.21 ± 3.26	20.34 ± 2.30	0.19	0.06_0.76
SJ (cm)	36.58 ± 3.95	36.31 ± 3.85	35.34 ± 3.99	0.04*	−0.06_0.24
CMJ (cm)	39.01 ± 2.46	39.50 ± 2.44	39.15 ± 2.31	0.89	0.25_0.14

Data are represented as means ± SDs. YYIR1: Yo-Yo intermittent recovery test level 1. RSSA: Repeated Shuttle Sprint Ability. [Lac]_*RSSA*_: Lactate concentration was measured after the Repeated Shuttle Sprint Ability- test. SJ: Squat Jump, CMJ: Countermovement Jump. *Significant difference between T3-T2 and T3-T1 with *p* < 0.05. £Significant differences between T2 and T1 with *p* < 0.05.

Team performance is illustrated in Table 5. Statistically significant differences were found between the congested and non-congested period. The percentage rate of winning matches amounted to 33% during the non-congested and 20% during the congested period (*p* < 0.001, *Z* = −4). Match accumulated time was higher in the congested period as compared to the non-congested period (*p* < 0.001, *Z* = 4).

**TABLE 5:** Performance of the soccer team during the congested and non-congested period.

Periods	Matches played	Win (%)	Draw (%)	Lost (%)	Matches accumulated- time (minutes)
From T1 to T2 non-congested period	6	33	0	67	540
From T2 to T3 congested period	10	20	0	80%	900

### Hormonal Data

The C levels were not significantly different during the different periods of training. On the other hand, T was significantly lower (*p* = 0.03, ES_3__–__2_ = −0.51, ES_3__–__1_ = 0.51) in T3 (5.08 ± 1.83) when compared to T2 (6.14 ± 2.30) and T1 (6.14 ± 2.19). The T/C ratio was significantly lower in T3 when compared to T2 and T1 (*p* = 0.017, ES_3__–__2_ = −1.1, ES_3__–__1_ = −1.07). The T level and T/C ratio were not significantly different in T2 as compared to T1 (Table 6).

**TABLE 6:** Changes of hormonal parameters during the 12 weeks training period.

Hormonal data	T1	T2	T3	*p*-Value	ESES_3–2 –_ ES_3–1_
Cortisol (ng/ml)	135.75 ± 15.19	134.56 ± 14.31	135.75 ± 10.58	0.89	0.09–0.0
Testosterone (ng/ml)	6.14 ± 2.30	6.14 ± 2.30	5.08 ± 1.83	0.03*	−0.51–−0.51
T/C ratio	0.044 ± 0.016	0.046 ± 0.019	0.03 ± 0.01	0.017*	−1.1–−1.07

Data are represented as means ± SDs. T/C: Testosterone/Cortisol. *Significant differences between T3-T2 and T3-T1 with *p* < 0.05.

### Psychological Data

Changes in scores from the POMS questionnaire at different time points are presented in Table 7. The vigor score assessed by the POMS questionnaire was significantly lower in T3 after the congested period than in the other time points (*p* = 0.002, ES_1__–__2_ = 0.31, ES_2__–__3_ = −1.25). Moreover, the fatigue score increased in T3 as compared to T2 and T1 (*p* = 0.002, ES_1__–__2_ = 0.43, ES_2__–__3_ = 0.81). The tension score increased progressively during the different competitive periods compared with T1 (*p* = 0.002, ES_1__–__2_ = 1.1, ES_2__–__3_ = 0.20). The anger score increased significantly (*p* = 0.03, ES_1__–__2_ = 0.47, ES_2__–__3_ = 0.33) from T1 to T2 and from T2 to T3. There were no alterations in the other psychological scores (depression and confusion). Finally, the Total Mood Disturbance increased significantly from T1 to T3 (i.e., T1 < T2 < T3; *p* = 0.001, ES_1__–__2_ = 0.91, ES_2__–__3_ = 1.10).

**TABLE 7:** Changes in psychological parameters.

Psychological scores	T1	T2	T3	*p*-Value	ESES_1–2 –_ ES_2–3_
Tension (A.U.)	9.31 ± 1.74	11.87 ± 2.75	12.37 ± 2.08	0.002*£	1.10_0.20
Depression (A.U.)	8.68 ± 1.35	10.18 ± 2.50	9.37 ± 1.92	0.08	0.78_−0.32
Anger (A.U.)	9.12 ± 2.12	10.18 ± 2.42	11.00 ± 2.53	0.03*£	0.47_0.33
Fatigue (A.U.)	9.12 ± 1.58	10.06 ± 2.72	12.31 ± 2.79	0.002*	0.43_0.81
Confusion (A.U.)	8.06 ± 2.04	8.75 ± 2.93	9.43 ± 2.55	0.35	0.27_0.24
Vigor (A.U.)	18.75 ± 2.38	19.43 ± 3.88	14.93 ± 3.41	0.002*	0.31_−1.25-
Total mood disturbance (A.U.)	25.56 ± 7.38	32.12 ± 6.90	39.06 ± 5.63	0.001*£	0.91_1.1

*Significant differences between T3 and T2 and between T3-T1 with *p* < 0.05. £Significant difference between T2 and T1 with *p* < 0.05. A.U.: Arbitrary units.

### Relationships Between Hormonal Parameters and Physical Fitness

Only a few significant correlations were found between hormonal data and physical fitness during the non-congested period (Table 8). The Δ% of testosterone correlated with Δ%RSSA_best_ (*r* = −0.7, *p* = 0.02). In addition, there was a significant relationship between Δ% of T/C parameters and Δ% of RSSA_best_ (*r* = −0.55, *p* = 0.02).

**TABLE 8:** Relations between hormonal parameters, and psychological parameters with measures of physical fitness.

	Non-congested period (T1–T2)					Congested period of match play (T2–T3)				
	YYIR1	RSSA mean	RSSA best	SJ	CMJ	YYIR1	RSSA mean	RSSA best	SJ	CMJ
**C**										
*r*	–0.4	0.01	–0.3	0.008	0.11	–0.15	0.3	–0.4	–0.32	0.22
*p*	0.07	0.9	0.14	0.97	0.12	0.62	0.1	0.12	0.91	0.49
**T**										
*r*	0.13	0.20	–0.7	0.18	0.21	0.18	–0.1	–0.3	0.31	0.16
*p*	0.60	0.43	0.02*	0.48	0.33	0.44	0.64	0.2	0.22	0.54
**T/C**										
*r*	0.12	0.23	–0.55	0.21	0.28	0.96	0.93	–0.1	0.10	–0.43
*p*	0.64	0.37	0.02*	0.42	0.32	0.72	0.75	0.6	0.55	0.11
**TMD**										
*r*	–0.2	0.3	–0.2	0.1	–0.7	–0.6	0.13	0.58	–0.55	0.18
*p*	0.3	0.1	0.2	0.6	0.7	0.02*	0.58	0.01*	0.01*	0.62

*Significant correlation between parameters. TMD: Total Mood Disturbance. YYIR1: Yo-Yo Intermittent Recovery test level 1. RSSA: Repeated Shuttle Sprint Ability. SJ: Squat Jump, CMJ: Countermovement Jump. C: Cortisol. T: Testosterone. T/C: Testosterone/Cortisol.

### Relationships Between Hormonal Parameters and Psychological Parameters

Several significant correlations were identified between hormonal parameters and psychological markers (POMS) during the congested period of match play (Table 9). There was a positive correlation between Δ% of total mood disturbance and Δ% of testosterone (*r* = 0.53; *p* = 0.03) and between Δ% of total mood disturbance and Δ% of T/C (*r* = 0.50, *p* = 0.04).

**TABLE 9:** Relations between hormonal parameters and psychological parameters.

	From T1 to T2 Non-congested period			From T2–T3 Congested-Period		
	Cortisol	Testosterone	T/C	Cortisol	Testosterone	T/C
**TMD**						
*r*	0.03	0.31	0.33	0.10	0.53	*r* = 0.50
*p*	0.89	0.21	0.19	0.65	0.03*	*p* = 0.04*

*Significant correlation between parameters. T/C: Testosterone/Cortisol. TMD: Total Mood Disturbance.

### Relationships Between Percentage Change of Psychological Parameters and Physical Fitness

**FIGURE 2:**
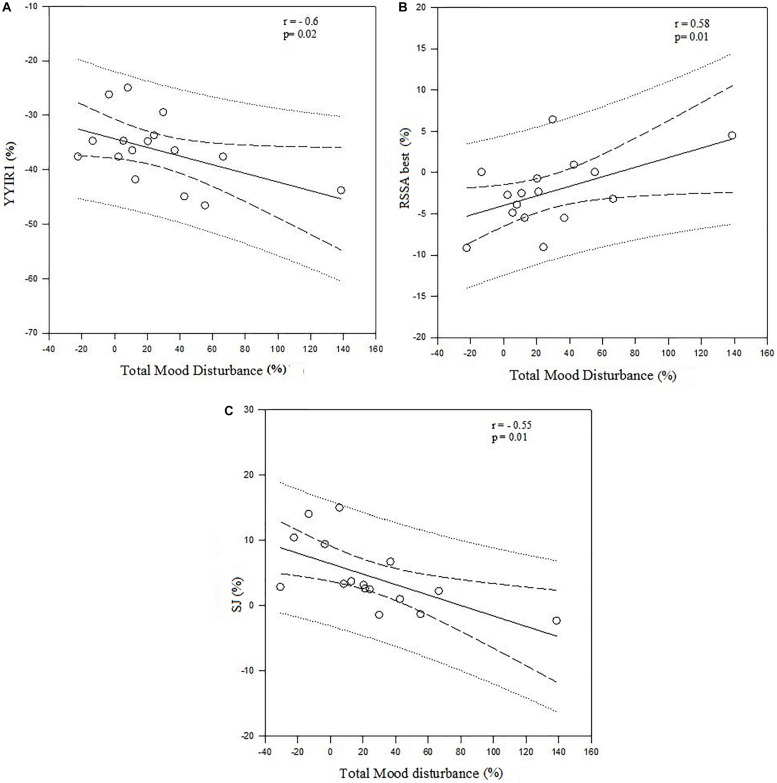
**(A)** Correlation between Δ% Total Mood Disturbance and Δ%YYIR1 during the congested period of match play. YYIR1: Yo-Yo Intermittent Recovery test level 1. **(B)** Correlation between Δ% total mood disturbance and RSSAbest during the congested period of match play. RSSA: Repeated Shuttle Sprint Ability test. **(C)** Correlation between Δ% total mood disturbance and SJ during the congested period of match play. SJ: Squat Jump.

No significant relationship was found between Δ% of total mood disturbance and Δ% of physical fitness during the normal competitive period. Of note, we observed a significant relationship between Δ% of total mood disturbance and Δ% of physical fitness during the period of congested match play (*r* = −0.6; *p* = 0.02; *r* = 0.58, *p* = 0.01; *r* = −0.55, *p* = 0.01, respectively, for YYIR1, RSSA_best_ and SJ) (Figures 2A–C).

### Relationships Between Percentage Change of Hormonal Parameters, Psychological Parameters, Physical Fitness and Workload Parameters

Table 10 summarizes relationships between percentage change of hormonal parameters, psychological parameters, physical fitness, and workload parameters during the study. We observed that changes in testosterone from T1 to T2 were related to workload (*r* = 0.6, *p* = 0.008). The Δ% of T/C ratio was related to workload from T1 to T2 (*r* = 0.62, *p* = 0.01). Also, the change in cortisol was related to monotony from T2 to T3 (*r* = 0.51, *p* = 0.01). In the non-congested period from T1 to T2, there was a significant relationship between training workload, monotony and strain, with physical fitness in RSSA_mean_ (*r* = 0.52, *p* = 0.03; *r* = 0.62, *p* = 0.01; *r* = 0.62, *p* = 0.009, respectively).

**TABLE 10:** Relations between percentage change of hormonal parameters, psychological parameters, and physical fitness and workload parameters.

	From T1 to T2 non-congested period			From T2 to T3 congested period		
	TRIMP	Monotony	Strain	TRIMP	Monotony	Strain
**Cortisol**						
*r*	0.37	0.25	0.32	0.19	0.51	0.27
*p*	0.12	0.35	0.22	0.42	0.01*	0.27
**Testosterone**						
*r*	0.66	0.33	0.44	–0.13	–0.12	–0.1
*p*	0.008*	0.20	0.08	0.5	0.6	0.4
**T/C**						
*r*	0.62	0.39	0.47	–0.21	–0.23	–0.25
*p*	0.01*	0.19	0.06	0.33	0.35	0.29
**TMD**						
*r*	0.06	0.15	0.15	0.11	0.25	0.11
*p*	0.79	0.56	0.56	0.63	0.31	0.66
**YYIR1**						
*r*	0.04	0.04	0.28	0.08	0.06	0.05
*p*	0.86	0.8	0.28	0.74	0.8	0.8
**RSSA Mean**						
*r*	0.52	0.62	0.62	0.02	–0.1	–0.04
*p*	0.03*	0.01*	0.009*	0.93	0.5	0.86
**RSSA best**						
*r*	–0.29	–0.07	–0.1	0.1	–0.09	0.08
*p*	0.26	0.77	0.70	0.69	0.71	0.7
**SJ**						
*r*	0.03	0.11	0.07	0.02	0.17	0.04
*p*	0.9	0.66	0.79	0.94	0.51	0.86
**CMJ**						
*r*	0.15	0.04	0.08	0.14	0.44	0.28
*p*	0.57	0.88	0.76	0.59	0.08	0.28

TRIMP: Training Impulse. T/C: Testosterone/Cortisol ratio. YYIR1: Yo-Yo intermittent recovery test level 1. RSSA: Repeated Shuttle Sprint Ability. SJ: squat jump. CMJ: Countermovement Jump. TRIMP: Training Impulse. TMD: Total Mood Disturbance. *Significant correlation between parameters.

## Discussion

**FIGURE 3:**
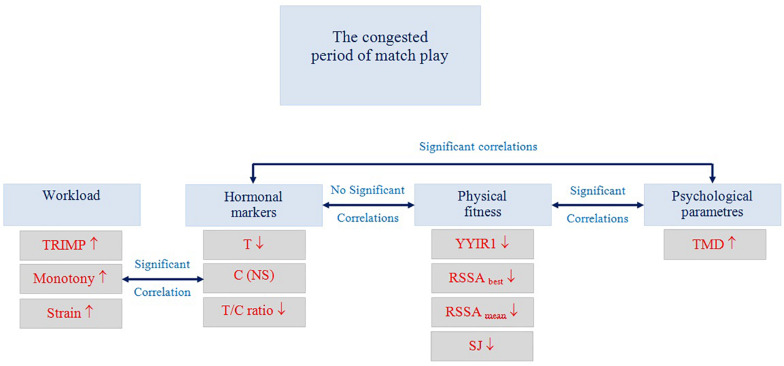
Summary of the main findings of this study. TRIMP: Training Impulse. C: Cortisol. T: Testosterone. T/C: Testosterone/Cortisol ratio. YYIR 1: Yo-Yo intermittent recovery test level 1. SJ: Squat Jump. TMD: Total mood disturbance.

This study examined elite soccer players’ fluctuations in hormonal, psychological, and physical fitness parameters during the competitive period of the season, which included a period of congested match play. In addition, the relationships among the various parameters were measured. The results showed significant alterations in hormonal, psychological and physical fitness parameters during the congested period of match play. However, while the changes observed in hormonal parameters were not related to physical fitness performance, the changes in psychological parameters were related to performance declines in physical fitness (Figure 3).

### Hormonal Markers

In this study, plasma C concentrations were unchanged during the 12-week competitive period and were not altered when players were exposed to the period of congested match play. This result is in agreement with data reported by Moreira et al. (2016) who investigated the subsequent impact of a congested period of match play (7 matches played over a 7-day period) on resting hormonal parameters in elite young soccer players from a reference team. Given the current results and those reported by Moreira et al. (2016), it can be speculated that congested periods of different density do not significantly affect the hypothalamic-pituitary-adrenal axis responses of players, when considering their resting C values. The absence of a significant change in resting C concentration may be, at least in part, attributed to the high fitness level of the players and to their habitual high daily and weekly training volume over the season. Further studies should verify the impact of congested periods of higher densities on hormonal responses.

Conversely, the current results disagree with the results of Handziski et al. (2006) who showed an increase in plasma C concentrations after the end of the competitive period. Differences between studies can be partially explained by distinct experimental protocol designs and participants’ characteristics. For example, the study of Handziski et al. (2006) and the current study analyzed the soccer season at different time points (e.g., half-season and 12 weeks competitive periods). In addition, the different degree of competition during the in-season might also explain the differing results. Previously, Silva et al. (2011) showed a 43.2% increase in plasma C levels in professional soccer players after a 12-week training program. The authors suggested that the increment of training intensity played an important role in cortisol increases. It is suggested that an increase in training intensity is the determining factor behind increases in C concentrations (Minetto et al., 2008). This can be explained by a hyper responsiveness of the HPA axis due to a physiological adaptation of the neuro endocrine system to training (Hackney, 2006). However, it is reasonable to suggest that the practical usefulness of using this approach to assess hormonal changes during highly intensive competition periods may be limited.

The C response to the competitive period during a congested schedule should be viewed with caution. In the current study, only resting plasma concentrations were examined, and on a limited basis (i.e., three-time points). Some authors have suggested that evaluating hormonal responses to exercise rather than resting may provide a better scenario for regarding hormonal changes. For instance, during training, or after a competitive match, or during states of non-functional overreaching (Meeusen et al., 2004; Hough et al., 2013; Silva et al., 2013), including the potential influence of circadian rhythms, and competitive vs. training workloads (Fernandez-Fernandez et al., 2015).

In contrast to C responses, a significant decrease in T concentrations was observed after the congested period of match play. The current results suggest that the higher number of matches played in the last 6 weeks (10 matches) potentially affected resting T concentrations. Thus, the accumulated fatigue potentially caused by the congested competitive schedule seems to lead to a decrement in T. Previous research found that T concentrations were related to tiredness and the magnitude of fatigue (Filaire et al., 2001; Maso et al., 2004; Silva et al., 2014). Maso et al. (2004) suggested that a low T level could be used as a marker of fatigue and might reflect the residual effect of previously accumulated fatigue, which could be linked to insufficient recovery intervals. In addition, Moreira et al. (2016) showed a decrease in T concentration measured after a congested match period (7 matches played in 7 days). Moreover, Filaire et al. (2001) showed a decrease in salivary T concentration measured after 7 weeks of high-intensity training. Based on previous results reported by Filaire et al. (2001) and Moreira et al. (2016), it appears that the T responses observed in the current study are associated with the accumulated fatigue generated during the congested period of match play.

In the present study, the soccer players showed significant reductions in the T/C ratio only after a period of congested match play, which is linked to the lowering in basal T levels. In fact, the decrease in T/C ratio of elite soccer players was accompanied by a reduction of physical performance. According to Gorostiaga et al. (2004), an impairment of physical performance could be accompanied by lower T/C values. A reduction in the T/C ratio is generally considered to be an indicator of anabolic-catabolic balance and it has previously been considered a useful tool for diagnosing overtraining (Urhausen et al., 1995). In the current study, it is possible that the decreased T/C ratio could mainly be explained by fatigue, which occurred after the heavy training load and the increased frequency matches played during the last 6-weeks of the competitive period. In addition, TRIMPs, monotony and strain values during the period of congested match play were higher compared with the non-congested period. This means that alterations in hormonal markers could be explained by the chronic effects of the last 6 weeks of competition. However, the average match exposure in our study was 1 match a week during the non-congested periods and 1.67 matches per week during periods of congested match play. In addition, match-accumulated time during the congested period was very high (=900 min in 6 weeks) compared to that of the non-congested period (=540 min in 6 weeks). This indicates that the higher match time exposures of elite soccer players during the last 6 weeks of competition could partially explain the observed deterioration in performance and hormonal changes. Thus, our data suggests that the accumulated match time during the competitive period of the season is associated with physical and hormonal parameters. That is, elite players presented a significant decrease in physical fitness (YYIR1, RSSA, and SJ) after a period with more match exposure.

### Mood State Changes

In the present study, the included elite players presented a typical “iceberg profile” after the 12-week competitive period (Morgan, 1980). In the iceberg profile, the athletes score below the 50th percentile with regards to tension, depression, anger, fatigue and confusion, and above the 50th percentile in terms of vigor. Previous data regarding the response to POMS assessment during 12 weeks competitive periods have suggested that negative changes of the mood state such a tension, fatigue and anger are a sign of staleness (i.e., a term typically used in psychology to represent overtraining) (O’Connor et al., 1989). Moreover, the positive score (vigor) of the POMS questionnaire was reduced, and the negative scores (fatigue, tension and anger) and the total mood disturbance, were increased after the congested period. In addition, Anger, tension scores and Total Mood disturbance increased during the non-congested periods. Moreover, it was hypothesized that players’ mood was affected by the greater training load applied along with the high number of matches played during the period of congested match play. In addition to workload influences, our study showed that players’ mood was affected by their performance and vice versa. During both, the congested and non-congested period, increased tension, anger, and decreased vigor coincided with poor performance. More specifically, the percentage rate of matches won fell below 50% for all played games (33% during the non-congested period versus 20% during the congested period). In this regard, Hoffman et al. (1999) showed when the team results are poor, the mood state vigor was reduced. As the teams’ results improved (a greater winning percentage), vigor returned to its normal level. In agreement with Filaire et al. (2001), who showed that the tension and depression score increased during the period of the season when the team winning percentage fell below the 50% for game played.

### Relationships Between Hormonal Parameters and Physical Fitness

In this study, no significant correlations were observed between hormonal parameters and players’ performance in the YYIR1, RSSA, SJ and CMJ tests, with only negative correlations observed between T, T/C ratio and RSSA_best_ during the non-congested period. These findings are in agreement with previous reports showing negative associations between changes in performance and T/C (Gorostiaga et al., 2004), and improved performance with maintenance of higher T/C values (Kraemer et al., 2004). In the current study, the decrease in physical fitness after the congested period of match play may be interpreted by a decrease in plasma T levels and T/C values. The observed-congested period of match play induced decrease in testosterone concentrations reflect decreased anabolic activity (Kraemer et al., 2004). Hence, Muscella et al., 2019 showed that the increased serum testosterone level together with serum cortisol decrement, would suggest a strong intensification of anabolic androgenic activity in young soccer players. It can reasonably be supposed that the alteration in testosterone concentration might affect explosive-type performance. In fact, positive associations between testosterone concentration and greater power performance were observed in young soccer players (Muscella et al., 2019). In addition, Hammami et al. (2017) demonstrated a positive correlation between T and T/C with physical fitness in SJ, CMJ and YYIR1 in elite soccer players. In contrast, Silva et al. (2013) have shown that a higher match exposure in the midseason was associated with higher C levels, a lower T/C, and better physical performances. These differences in results suggest that the analysis of the T/C ratio should be used with caution as noted by others (McLellan et al., 2010). Our results further suggest that the decrease in T/C ratio noted in T3 after the congested period of match play may reflect tiredness or a state of fatigue. To determine more precisely the influence of fatigue on these hormonal variations, it is necessary to address these two variables. To do this, we examined potential correlations between the total mood disturbance and hormonal data.

### Relationships Between Mood State With Hormonal Parameters and Physical Fitness

In our study, C concentrations were not related to total mood disturbance during different competitive periods. In contrast, T concentrations showed a relationship with Total mood disturbance during the congested period suggesting the involvement of mood changes on T responsiveness. This is in accordance with previous studies (Maso et al., 2004; Elloumi et al., 2008). Moreover, we found a significant positive correlation between the T/C ratio and the Total mood disturbance. This suggests that it is more useful to follow variations in T (an anabolic hormone) than variations in C (a catabolic hormone) to determine the degree of tiredness and change in Mood state (Maso et al., 2004). In addition, there appears to be a negative relationship between physical fitness and total mood state during a period of congested match play. Thus, the increase of total mood disturbance was associated with physical fitness declines. These results cannot, however, be used to indicate whether changes in physical fitness are affected by changes in mood (T-score on fatigue, anger and tension increased and T-score on vigor decreased) or vice-versa. Our results confirm that physical performance depends simultaneously on physical and psychological characteristics. Thus, it seems that the participation in a high number of matches during the congested period induces psychological stress on soccer players. Most likely due to the need for sustained physical and cognitive demands during every match. In addition, anger, tension and total mood disturbance were observed to increase, during the non-congested period, but these changes appeared to be independent of physical fitness. YYIR, and best RSSA increased after the non-congested period. It could be assumed that the changes in mood states observed during this period are related to other factor than staleness. The financial problems experienced by the players in this study may have resulted in the observed changes in the mood traits anger and tension. However, soccer players were still able to perform during physical test well regardless of these changes in mood during the non-congested period. Moreover, this result could be explained by the professionalism of these players who need to show their physical ability to coach in order to confirm their selection during the matches.

In this study, we showed a positive correlation between monotony and C variations during the congested period of match play. In addition, changes of T concentrations and T/C ratio showed a significant relationship with workload during the non-congested period. These results confirm that some hormones are sensitive to competitive periods and soccer training programs which is in agreement with the study of Silva et al. (2011) who showed that increased C and decreased C levels are related to increases in both, training intensity and volume. Thus, the hormonal levels can be used as potential markers for variations of volume and intensity of training. While a few studies exist that assessed hormonal variation, mood changes and performance data in athletes (Hoffman et al., 1999; Filaire et al., 2001). Our study additionally examined the interplay between these factors. Findings from this study suggest that the observed changes in hormonal parameters during the congested period of match play were related to psychological but not physical fitness parameters. We acknowledge that the use of the POMS questionnaire is limited in team sports. Players from the starting line-up may exhibit a more positive mood state during the competitive period compared with substitutes because they know that they will get match time. Thus, different motivational levels of the team players may have affected the outcomes of the POMS questionnaire.

This study has a number of limitations that warrant discussion. First, the study cohort was composed of a single team only which is why findings are specific to the examined cohort and environment. Caution is needed when transferring our findings to female soccer or male soccer of different expertise level. Second, the sample size is rather small and the number of measurement points is limited which is why more research is needed to verify our findings.

## Conclusion and Practical Applications

This study analyzed hormonal (plasma C concentration, plasma T concentration), psychological, physical, workload, and physical fitness parameters in elite soccer players, and their relationships with changes in training and match exposure (congested and non-congested periods of match play). The results of this study showed significant changes in various hormonal, psychological and physical fitness parameters during the final competitive period of the season (congested match play). Our results suggest that the congested period of match play alters various parameters in elite soccer players. While the observed changes in hormonal parameters were not related to declines in physical fitness, those in psychological parameters were. Our findings lead us to suggest the use of the POMS questionnaire as an appropriate and simple monitoring tool for identifying performance decrements in players exposed to high competitive loads during the congested period. This recommendation may provide useful information for coaches and medical staff for better monitoring workload and physical fitness to manage players’ work-strain ratio to avoid overreaching – overtraining, and to reduce the risk of sustaining injuries.

## Data Availability Statement

The raw data supporting the conclusions of this article will be made available by the authors, without undue reservation.

## Ethics Statement

The studies involving human participants were reviewed and approved by the Ethics Committee of the Scientific Council of the University of Rennes 2, France and the medical staff of the soccer club (Jeunesse Sportive Kairouannaise, JSK). The patients/participants provided their written informed consent to participate in this study.

## Author Contributions

KS, AB, and HZ conceived and designed the research, and conducted the experiment. KS and AB analyzed the data. KS, AB, DB, UG, GD, AH, BB, TP, and HZ wrote and revised the manuscript. All authors read and approved the manuscript.

## Conflict of Interest

GD is employed by the company Real Madrid FC (Madrid, Spain). The remaining authors declare that the research was conducted in the absence of any commercial or financial relationships that could be construed as a potential conflict of interest.
